# Identifying Apoptosis-Related Transcriptomic Aberrations and Revealing Clinical Relevance as Diagnostic and Prognostic Biomarker in Hepatocellular Carcinoma

**DOI:** 10.3389/fonc.2020.519180

**Published:** 2021-02-18

**Authors:** Jinyu Zhu, Bufu Tang, Xiuling Lv, Miaomiao Meng, Qiaoyou Weng, Nannan Zhang, Jie Li, Kai Fan, Liyun Zheng, Shiji Fang, Min Xu, Jiansong Ji

**Affiliations:** ^1^ Key Laboratory of Imaging Diagnosis and Minimally Invasive Intervention Research, Lishui Hospital, School of Medicine, Zhejiang University, Lishui, China; ^2^ Department of Radiology, Second Affiliated Hospital, School of Medicine, Zhejiang University, Hangzhou, China; ^3^ Department of Radiology, The Fifth Affiliated Hospital of Wenzhou Medical University, Lishui, China

**Keywords:** apoptosis, hepatocellular carcinoma, overall survival, relapse-free survival, diagnosis, prognosis

## Abstract

In view of the unsatisfactory treatment outcome of liver cancer under current treatment, where the mortality rate is high and the survival rate is poor, in this study we aimed to use RNA sequencing data to explore potential molecular markers that can be more effective in predicting diagnosis and prognosis of hepatocellular carcinoma. RNA sequencing data and corresponding clinical information were obtained from multiple databases. After matching with the apoptotic genes from the Deathbase database, 14 differentially expressed human apoptosis genes were obtained. Using univariate and multivariate Cox regression analyses, two apoptosis genes (BAK1 and CSE1L) were determined to be closely associated with overall survival (OS) in HCC patients. And subsequently experiments also validated that knockdown of BAK1 and CSE1L significantly inhibited cell proliferation and promoted apoptosis in the HCC. Then the two genes were used to construct a prognostic signature and diagnostic models. The high-risk group showed lower OS time compared to low-risk group in the TCGA cohort (P < 0.001, HR = 2.11), GSE14520 cohort (P = 0.003, HR = 1.85), and ICGC cohort (P < 0.001, HR = 4). And the advanced HCC patients showed higher risk score and worse prognosis compared to early-stage HCC patients. Moreover, the prognostic signature was validated to be an independent prognostic factor. The diagnostic models accurately predicted HCC from normal tissues and dysplastic nodules in the training and validation cohort. These results indicated that the two apoptosis-related signature effectively predicted diagnosis and prognosis of HCC and may serve as a potential biomarker and therapeutic target for HCC.

## Introduction

Primary liver cancer, 85–90% of which are hepatocellular carcinomas (HCCs), represents a major health burden being the sixth most commonly diagnosed cancer and the fourth leading cause of cancer death worldwide, and the incidence and mortality of HCC are dramatically increasing yearly (1–3). There are multiple management strategies used for treating HCC, each accompanied by different risks and rewards. For instance, the surgical options available are partial liver resection, percutaneous ablation, TACE, and either living or cadaveric donor transplantation, while radiation therapies are used for advanced HCC (4, 5). However, the relapse rates and adverse outcome rates of HCC remain high, and the 5-year relapse-free survival (RFS) and overall survival (OS) rates for HCC are still poor (6, 7). Therefore, reliable prognosis markers are strongly needed to establish dependable prognosis models and make accurate survival predictions, which may help to specify individualized treatment options. The molecular pathogenesis of HCC is very complex, with multiple genetic variations, such as mutations and deletions of genes, and alterations in key molecular regulatory pathways (8). Based on this status quo, it is very urgent to explore new molecular markers from the molecular mechanism level to improve the accuracy of prognosis and survival prediction of HCC.

Apoptosis, as an important and tightly governed process of programmed cell death (PCD) with distinct biochemical and genetic pathways, involving the genetically determined elimination of cells (9). Apoptosis not only occurs as a fundamental homeostatic mechanism in the maintenance of normal cell turnover in tissues during development and aging (10), but previous research has revealed that dysfunctional apoptosis (too little or too much apoptosis or apoptosis occurring in the wrong place/time) also leads to variable diseases and multiple types of cancer (11). Many human diseases can be directly or indirectly attributed to the disorder of the apoptotic mechanism, which, triggered by abnormalities in the apoptosis pathway, lead to the excessive accumulation of cells or the loss of the disease (12). Previous studies have reported that the apoptotic pathway is important in controlling the critical processes of HCC. For instance, Changqing Su et al. (13) revealed that survivin, as a member of the inhibitor of apoptosis protein (IAP) family, is highly expressed in HCC, and its overexpression can promote cancer cell proliferation, inhibit cancer cell apoptosis, reduce the sensitivity of cancer cells to radiotherapy and chemotherapy, and ultimately affect the prognosis of patients with HCC. Few studies have explored the association between apoptotic genes and the diagnosis and prognosis of HCC through high-throughput sequencing of biomarkers.

In this study, we aimed to explore key apoptosis-related genes that can help predict the diagnosis and prognosis of HCC more accurately as new molecular markers. We explored the differences in mRNA expression between HCC tissues and adjacent noncancerous tissues from the TCGA database to determine the apoptotic-related genes involved in the prognosis of liver cancer. Functional enrichment analysis of these apoptosis-related genes was performed. Through univariate and multivariate Cox regression analysis, we eventually identified and constructed a prognostic model of two apoptosis-related genes, which contained BAK1 and CSE1L. Knockdown of BAK1 and CSE1L were showed to inhibit the proliferation of HCC cells, and significantly promote the apoptosis of HCC. And two diagnostic models were constructed by the same genes to distinguish HCC from normal samples and dysplastic nodules. Moreover, a predictive nomogram based on survival was constructed and evaluated to improve the prognostic and survival prediction accuracy of HCC patients and help clinicians make more useful clinical decisions. We also validated the expression patterns of the two genes in the Gene Expression Omnibus (GEO), TCGA cohort, ICGC cohort, and online databases including UALCAN and Kaplan-Meier Plotte. GSEA analysis was performed to explore the underlying mechanism of the two genes in HCC.

## Materials and Methods

### Apoptosis-Related Genes

Fifty-four apoptosis genes were extracted from the Deathbase (http://deathbase.org), which contains a series of proteins and corresponding coding genes of various species involved in cell death.

### Selection of HCC Associated RNA-Sequencing Data

The RNA sequence data (Illumina HiSeq RNA-Seq platform) and corresponding clinical information of liver hepatocellular carcinoma (LIHC) patients were obtained from the Cancer Genome Atlas (TCGA) data portal. The LIHC cohort contained 370 HCC samples and 50 normal tissue samples as of July 8, 2019. Considering that the RNA sequence data were downloaded from TCGA, they are open to the public and are freely available, and this study strictly followed the access policy and publishing guidelines of the TCGA database, so there was no need to seek approval from the Ethics Committee for this research.

### Differentially Expressed mRNA Screening Between HCC and Adjacent Non-Cancerous Tissues

First, we matched the 54 apoptotic genes obtained from the Deathbase database with 19,677 annotated coding genes of HCC downloaded from the RNA-sequencing platform in the TCGA database, from which we obtained 45 human apoptosis genes associated with HCC. Then, for subsequent analysis, we used the limma. R package to identify 14 differentially expressed genes (DEGs) of the dataset with absolute log2 fold change (FC) >1 and adjusted P value <0.05.

### Functional Enrichment Analysis

The Database for Annotation, Visualization and Integration Discovery (DAVID) (https://david.ncifcrf.gov) was used to analyze The Gene Ontology (GO), and KOBAS database (kobas.cbi.pku.edu.cn) was used to perform Kyoto Encyclopedia of Genes and Genomes (KEGG) pathway enrichment analyses of differentially expressed HCC apoptosis-related genes. Adjusted P < 0.05 was considered to represent statistical significance.

### Construction of the Apoptosis-Related Prognostic Model

Next, we validated the correlation between OS of HCC patients and specific expression levels of each apoptosis-related gene by univariate and multivariate Cox regression analysis. In the univariate Cox regression analysis, a P value for a gene <0.05 was considered to represent statistical significance, and the gene was also considered to be significantly associated with the OS of HCC patients. Subsequent multivariate Cox regression analysis further screened two apoptosis-related genes that could be deemed crucial prognostic factors for evaluating survival, thereby ultimately constructing the optimal apoptosis-related prognostic signature. Prognostic risk scores based on two apoptosis-related genes were established by regression coefficients of the multivariate Cox regression model (β) and gene expression levels. Prognostic index (PI) = (β* expression level of BAK1) + (β* expression level of CSE1L). Setting the median of the PI as a cut-off value, 370 HCC patients were divided into a high-risk group and a low-risk group from the prognostic model.

### Build of the Apoptosis-Related Diagnostic Model

Logistic regression analysis was applied in building a diagnostic model to distinguish HCC from normal samples and dysplastic nodules. The P value is defined as the probability that patient may have HCC. In order to make the P value between 0 and 1, we performed logistic transformation on P and use logit(P) as the dependent variable, that is, Logit(P) = ln[p/(1−p)] = α+β_1_x_1_+β_2_x_2_. x_1_ represents the expression level of BAK1 and x_2_ represents the expression level of CSE1L. Based on the expression data of BAK1 and CSE1L in HCC patients and normal subjects from the TCGA database, the parameters are estimated, and the coefficients of the logistic regression equation are estimated using SPSS (version 24.0) to obtain the logit(P) equation. If P of a subject calculated based on the expression of BAK1 and CSE1L is between 0 and 0.5, it is classified as a healthy person, otherwise it is classified as a patient with HCC.

### Establishment and Validation of a Predictive Nomogram

To verify whether the predictive power of this apoptosis-related prognostic signature was independent of other clinical features of HCC patients, which included age, sex, weight, AFP, vascular tumor cell invasion, clinical stage, and pathological grade, we performed univariate and multivariate Cox regression analysis, setting the prognostic signature and clinical features as independent variables, and OS as the dependent variable. Bilateral P values <0.05 were considered to represent statistical significance. Then, the hazard ratio and 95% confidence interval (CI) were calculated for each clinical variable.

Based on these independent prognostic factors, a predictive nomogram (including important independent prognostic factors and calibration maps) was constructed using rms R software. The nomogram was then evaluated and validated by calibration and discrimination. In addition, a consistency index (C index) is calculated using a bootstrap method with 1,000 resamples to determine the distinction of the nomogram. Meanwhile, we used the C index, time-dependent receiver operating characteristic (ROC) analysis and decision curve analysis (DCA) to compare the prediction accuracy between this nomogram and other nomograms constructed by single individual prognostic factors. DCA is a novel tool for evaluating predictive models that quantifies the clinical applicability of nomograms by analyzing the clinical outcomes resulting from decisions made based on predictive nomograms and is of great value in determining alternative diagnostic and prognostic strategies (14). P value <0.05 was considered to represent statistical significance.

### Cell Culture and siRNA Treatment

The human hepatocellular carcinoma cell line and human L02 hepatocytes were obtained from the American Type Culture Collection (ATCC) (Manassas, VA, USA). Cells cultured in DMEM media supplemented with 2 mM L-glutamine, 100 U/ml (penicillin-streptomycin), and 10% fetal bovine serum (FBS) in 5% CO2 at 37°C in a constant temperature incubator. SK-HEP1 and SMMC-7721 was transfected 50 nM BAK1 and CSE1L siRNA in basic DMEM media for 6–8 h before change with DMEM media supplemented FBS and penicillin-streptomycin.

### Western Blot Analysis

Protein was collected from SK-HEP1 and SMMC-7721 cells using protein lysis buffer containing PMSF (Bio-Rad). The BCA method was used to determine the total protein concentration. After adding the loading buffer to the protein samples, heating the protein samples at 100°C for 5 min. Protein samples were separated using 10% sodium lauryl sulfate-polyacrylamide gel electrophoresis (SDS-PAGE) and then transferred to a PVDF membrane. Then 5% skim milk was formulated to block the blot for 1.5 h at room temperature on a shaker. The blots were incubated with specific primary antibodies on a shaker overnight at 4°C. Imprinted with horseradish peroxidase-conjugated secondary antibody and processed with ECL kit (Bio-Rad) to detect protein.

### RNA Preparation, Reverse Transcription, and qRT-PCR

Total RNA was extracted using the TRIzol^®^ reagent (Invitrogen, shanghai, China), and the cDNA was prepared by TransScript Top Green qPCR supermix (TransGen, Guangzhou, China) according to the manufacturer’s protocol. Relative gene expression was performed with RT-PCR detection system (Bio-Rad Laboratories, Inc., Hercules, CA, USA) with Real-Time PCR Master Mixes (TransGen, Guangzhou, China) according to the manufacturer’s protocol. β-actin was served as an endogenous control. The relative expression (fold change) of target mRNA was measured using the 2-ΔΔCT methodology. Each experiment was repeated three times independently.

### Cell Proliferation Assay

After transfecting BAK1 and CSE1L siRNA for 48 h, SK-HEP1 and SMMC-7721 were cultured in 96-well plates for 48 h, the cell number was 3,000/plate with 200 ul DMEM. Then CCK8 agent (Yeasen, Shanghai, China) was treated per plate and OD450 was analyzed by Micro plate spectrophotometer (Thermo Fisher Scientific, MA, USA). Edu assay was also performed to measure the cell proliferation. Edu agent was obtained from Ruibo company (Ruibo, Guangzhou, China) and the Edu assay is performed according to the manufacturer’s protocol.

### Flow Cytometry to Measure Apoptotic Cells

After transfecting BAK1 and CSE1L siRNA for 72 h, Flow cytometry was used to detect the apoptosis of SK-HEP1 and SMMC-7721 cells. Annexin and PI agent (Yeasen, Shanghai, China) was used to label the viable apoptotic cells and non-viable apoptotic cells according to the manufacturer’s protocol. Flow cytometry was used to measure the number of apoptotic cells. All samples were analyzed using NovoExpress installed with software.

### Statistical Analysis

Statistical analyses were performed using SPSS, version 22.0 (SPSS, Chicago, IL, USA). Values were expressed as the mean ± SD. Quantitative data in paired groups were determined using the Student’s t-test. One-way analysis of variance was performed for multiple group comparisons. A value of P < 0.05 indicated significant differences.

## Results

### Identification and Validation of the Key Apoptotic Genes Closely Related to OS in HCC Patients

**Figure 1:**
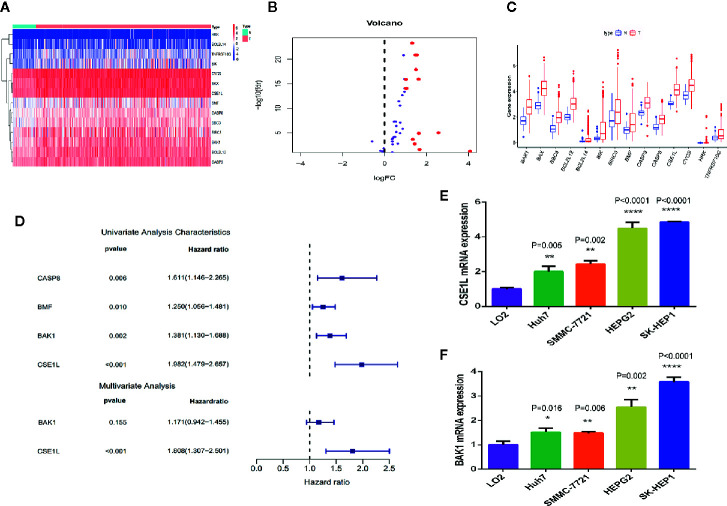
Differential expression analysis and identification of key apoptosis genes related to prognosis in HCC. **(A)** The heat map showed the expression levels of 14 apoptotic genes in HCC tissues and noncancerous tissues. **(B)** The histogram indicated the expression patterns of 14 apoptotic genes in HCC tissues and noncancerous tissues. **(C)** The volcano plot indicates that the 14 differentially expressed genes satisfy the condition of P < 0.05 and log FC > 1. **(D)** Univariate and multivariate Cox regression analysis performed to identify the genes closely associated with the prognosis of HCC patients. **(E, F)** qRCR was used to measure the mRNA level of CSE1L **(E)** and BAK1 **(F)** in human L02 hepatocytes and HCC cell lines. *P < 0.05; **P < 0.01; ****P < 0.0001.

By comparing the differentially expressed genes in mRNA expression profiles between HCC tissues (n = 370) and adjacent non-cancerous tissues (n = 50) and matching with apoptotic-related genes (**Table S1**), 14 differentially expressed human apoptotic genes (log FC>1 or log FC<-1, adjusted P < 0.05) adjusted for false discovery rate were obtained (**Figures 1A, B**). All the genes were overexpressed in the HCC tissues (**Figure 1C**). We then further explored the relevance of these differentially expressed apoptotic genes to the prognosis of HCC.

Next, we investigated the potential biological processes and pathways of these 14 differentially expressed apoptotic genes. We performed a GO function enrichment analysis of these genes through the DAVID database and finally identified 10 important GO terms (adjusted p < 0.05). The association between apoptotic genes and GO terms was visualized through the GOplot.R package (**Figure S1A**). According to the degree of gene function enrichment, the top five GO terms were “positive regulation of release of cytochrome c from mitochondria,” “positive regulation of apoptotic signaling pathway,” “regulation of apoptotic signaling pathway,” “positive regulation of mitochondrion organization,” and “regulation of release of cytochrome c from mitochondria.” Subsequently, we performed a KEGG pathway analysis concentrating on these genes through the KOBAS database and finally determined 26 statistically significant KEGG pathways (adjusted P < 0.05). We then visualized the association between apoptotic genes and KEGG through the enrichplot.R package (**Figure S1B**), and the top three important pathways among them were “apoptosis-multiple species,” “apoptosis,” and “platinum drug resistance.”

To analyze the association between these 14 differentially expressed apoptotic genes and OS in HCC patients, we performed a univariate Cox regression analysis and screened four apoptotic genes (CASP8, BMF, BAK1, CSE1L) closely associated with OS in HCC patients (P < 0.05) (**Figure 1D**). Then, through multivariate Cox regression analysis, two apoptotic genes (BAK1 and CSE1L) were identified to be significantly associated with OS in HCC patients. To explore the role of two apoptotic genes in HCC, qPCR assay was used to measure the mRNA of BAK1 and CSE1L in different cell lines. We found BAK1 and CSE1L level in HCC cells was obviously higher than in human L02 hepatocytes (**Figures 1E, F**).

### Identified the Tumoral Effect of Critical Apoptotic Regulators in Hepatocellular Carcinoma Cells

**Figure 2:**
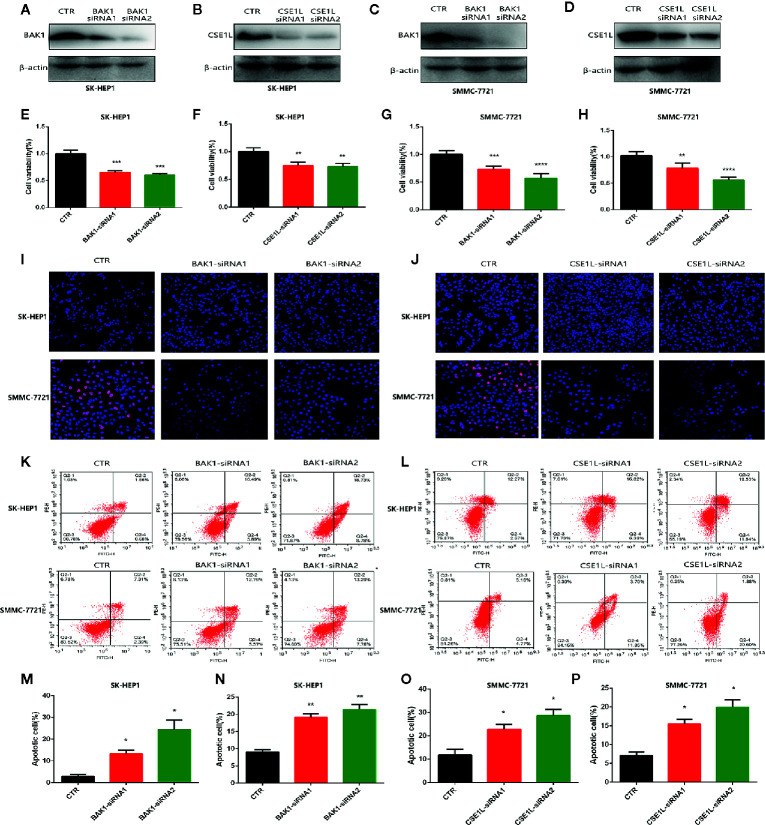
Measure the oncogenic effect of BAK1 and CSE1L in the HCC. **(A–D)** Western blot determined that expression of BAK1 and CSE1L was inhibited with BAK1 and CSE1L siRNA administration. **(E–H)** CCK8 assay showed BAK1 and CSE1L inhibition significantly suppressed HCC cells proliferation. **(I, J)** Edu assay indicated knock down BAK1 and CSE1L inhibited HCC cells proliferation, respectively. **(K, L)** Flow cytometry confirmed decreased BAK1 and CSE1L expression promoted HCC cells apoptosis in the HCC. **(M–P)** Quantitative statistical results of the effects of BAK1 and CSE1L expression on cell apoptosis. Data are shown as the mean ± SD of at least three independent experiments. *P < 0.05, **P < 0.01, ***P < 0.001, ****P < 0.0001.

To further investigate explore the oncogene role of the two critical apoptotic regulators in Hepatocellular Carcinoma cells. Then we knocked down the level of BAK1 and CSE1L in HCC cells including SMC-7721 and SK-HEP1. Western blot showed the expression of BAK1 and CSE1L was obviously inhibited with BAK1 and CSE1L siRNA administration (**Figures 2A–D**). The CCK assay showed BAK1 inhibition suppressed HCC cells proliferation (**Figures 2E, G**) and the anti-proliferation effect of CSE1L inhibition was similar to BAK1 knockdown in HCC cells (**Figures 2F, H**). In addition, Edu assay indicated BAK1 and CSE1L inhibition significantly suppressed HCC cells proliferation in SMC-7721 and SK-HEP1 cell (**Figures 2I, J**). Moreover, flow cytometry showed BAK1 and CSE1L suppression promoted cell apoptosis in SMC-7721 and SK-HEP1 cells (**Figures 2K, L**), and quantitative statistical results are presented in **Figures 2M–P**. The above results confirmed that BAK1 and CSE1L inhibited HCC cells apoptosis and were closely related to the HCC cells proliferation.

### Construction of the Apoptosis-Related Prognostic Signature and Evaluation of Its Predictive Performance of Prognosis in HCC Patients in TCGA Cohort

**Figure 3:**
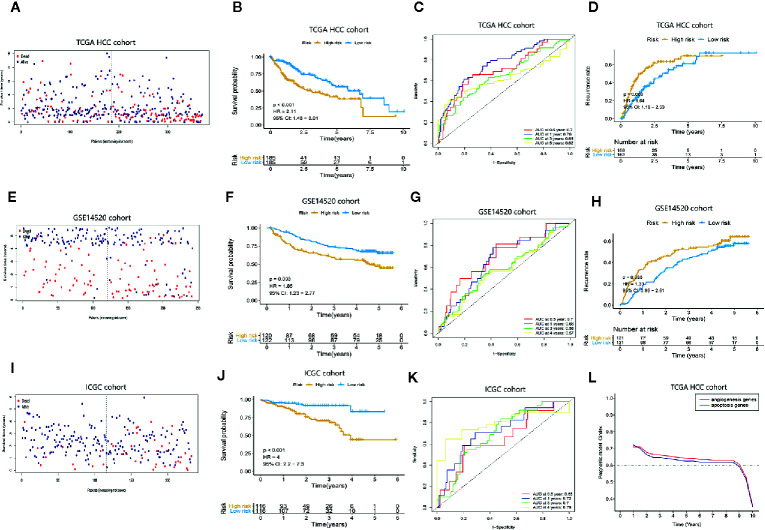
Assessment of the predictive performance of the apoptosis-related prognostic signature. Kaplan-Meier survival analysis, heat map and time-dependent ROC curves of the prognostic signature in the TCGA HCC cohort, GEO HCC cohort, and ICGC cohort. **(A, E, I) **The survival status of the HCC patients with different risk scores. **(B, F, J)** K-M curves indicate that OS in high-risk patients is significantly lower than in low-risk patients (P < 0.05). **(C, G, K)** Time-dependent ROC curve analysis of the apoptosis-related prognostic signature. **(D, H)** K-M curves showed the difference of recurrence rates between high-risk patients and low-risk patients. **(L)** The difference in predictive performance of the two prognostic models.

An apoptosis-related prognostic signature based on BAK1 and CSE1L was established. The predictive index (PI) = (0.157511873* the expression level of BAK1) + (0.592406034* the expression level of CSE1L). The median PI was set to the cut-off value, and all 370 HCC patients were divided into a high-risk group (n = 185) and a low-risk group (n = 185) (**Figure S2A**). The corresponding relationship between the patient’s risk score and survival status is shown in **Figure 3A**, revealing that patients with low-risk scores had better survival rates than those with high-risk scores (P < 0.05). The expression levels of BAK1 and CSE1L were different in HCC patients with different risk scores (**Figure S2B**). Using the HCC patients in the TCGA cohort, the K-M curve was used for survival analysis. The high-risk group showed lower survival time compared to low-risk group (P < 0.001, HR = 2.11) and the OS probabilities at 1 year, 3 years, and 5 years were 0.72, 0.48, and 0.37 in high-risk patients and 0.926, 0.73, 0.56 in low-risk patients (**Figure 3B**). The recurrence probabilities of high-risk patients were higher than those of low-risk patients (P = 0.003) (**Figure 3D**). The AUCs of the prognostic signature were 0.7, 0.76, 0.65, and 0.62 at 0.5 year, 1 year, 3 years, and 5 years, respectively (**Figure 3C**), suggesting that the prognostic signature has good predictive power.

### External Validation of the Predictive Power of This Apoptosis-Related Prognostic Signature for Prognosis in HCC Patients in the GEO Cohort and ICGC Cohort

To further confirm that the apoptosis-related prognostic signature has good predictive value in terms of prognosis in HCC patients, the prognostic signature was applied to the GSE14520 cohort (containing 243 tumor samples and 241 adjacent non-tumor samples) and ICGC cohort (including 243 tumor samples and 202 adjacent non-tumor samples). Using the same cut-off value obtained in the TCGA cohort, the GSE14520 cohort and ICGC cohort were divided into a high-risk group and a low-risk group (**Figures S2C, E**). **Figures 3E, I** show that patients with low-risk scores in this data set had better survival probabilities than those with high-risk scores, and these results are consistent with the TCGA cohort. The expression levels of BAK1 and CSE1L in patients with different risk scores are shown in **Figures S2D, F**. K-M curve analysis was performed on HCC patients in the GSE14520 cohort and ICGC cohort, the high-risk group showed lower survival time compared to low-risk group in GSE14520 cohort (P = 0.003, HR = 1.85) and ICGC cohort (P < 0.001, HR = 4) (**Figures 3F, J**). Moreover, high-risk patients showed higher recurrence rates in GSE14520 cohort (**Figure 3H**). The AUCs in the time-dependent ROC curve analysis reached 0.7, 0.66, 0.58, and 0.56 of GSE14520 cohort (**Figure 3G**) and 0.65, 0.72, 0.7, and 0.79 at of ICGC cohort (**Figure 3K**) at 0.5 year, 1 year, 3 years, and 4 years, suggesting that the prognostic signature has good predictive sensitivity and specificity. The result suggested the prognostic model can be applied to different platforms and has good clinical value in GSE14520 cohort and ICGC cohort. And compared with our previous research (15), we confirmed that the apoptosis-related prognostic model performed better prediction accuracy (**Figure 3L**)

### Hierarchical Analysis of Predictive Performance of OS in This Apoptosis-Related Prognostic Signature Based on Clinical Stage

**Figure 4:**
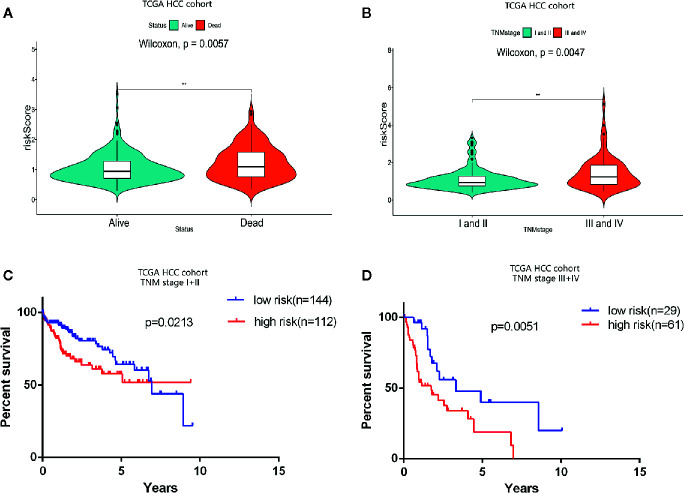
Prognostic analysis of the apoptosis-related prognostic signature according to clinical TNM stage. **(A)** Violin plot revealing significantly different risk scores between patients in stages I and II and patients in stages III and IV (**P < 0.01). **(B)** Violin plot revealing significantly different risk scores between alive patients and dead patients (**P < 0.01). **(C**, **D)** Kaplan-Meier (K-M) survival analysis of different clinical TNM stages.

Consistent with the apoptosis-related prognosis signature, the TNM stage was also significantly correlated with the prognosis and survival status of HCC patients. The survival status resulting from the progression of HCC indicated that the risk index of the dead patients was significantly higher than that of the alive patients (P = 0.004) (**Figure 4A**). And the prognostic risk index of patients in TNM stages III and IV was determined to be significantly higher than that of patients in stages I and II (P = 0.005) (**Figure 4B**). Hierarchical analysis was used to determine whether the prognostic performance of the prognostic signature was independent of clinical TNM stage. HCC patients in TCGA cohort were divided into two groups according to TNM stage: I, II, and III, IV. The hierarchical analysis authenticated that the prognostic signature was significantly associated with OS in the HCC cohorts of both groups (**Figures 4C, D**), indicating that the prognostic signature has good predictive performance for OS in HCC patients, which was independent of clinical TNM stage.

### The Apoptosis-Related Prognostic Signature Is Independent of Traditional Clinical Features in Predicting OS

**Figure 5:**
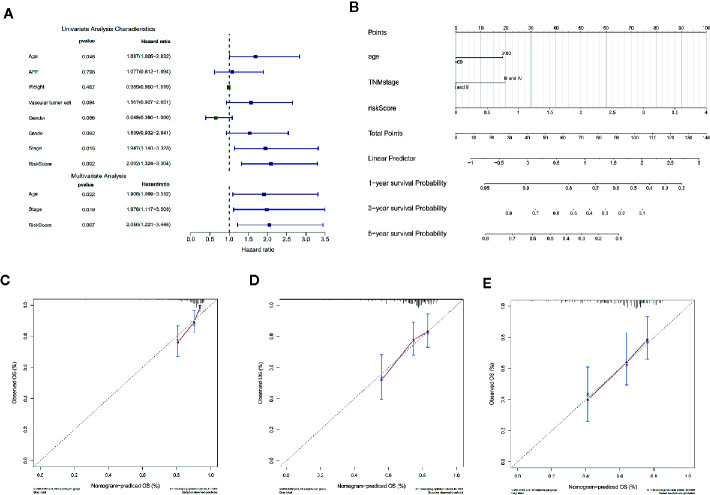
Cox regression analysis and nomogram incorporating three independent prognostic factors was constructed to predict OS in HCC patients. **(A)** Univariate and multivariate Cox regression analysis of the apoptosis-related prognostic signature and clinical features with overall survival (OS) in HCC patients (P < 0.05). **(B)** The total points were obtained by cumulatively adding the corresponding points of the independent prognostic factors on the point scale to predict the OS of the HCC patients at 1 year, 3 years, and 5 years in the nomogram. **(C–E)** The calibration curve to assess the predictive value of the nomogram in OS at 1 year, 3 years, and 5 years. The Y-axis represents the actual OS measured by K-M curve analysis, and the X-axis represents the OS predicted by the nomogram.

In order to assess the independent predictive value of this apoptosis-related prognostic signature, univariate and multivariate Cox regression analyses were used to analyze 370 HCC patients with intact clinical features from the TCGA cohort. Univariate Cox regression analysis revealed that age, clinical stage, and the apoptosis-related prognostic signature were independent prognostic factors associated with OS in HCC patients (P < 0.05) (**Figure 5A**) (**Table S2**).

For more accurate prediction of the prognosis and survival probability of HCC patients and to help clinicians make clinical decisions and develop personalized treatment plans for patients, we constructed a predictive nomogram (**Figure 5B**). Compared with age and clinical stage, the risk score of the prognostic signature had the greatest impact on total points, which is consistent with the results of multivariate Cox regression analysis. The C index of the nomogram was 0.6777 after 1,000 bootstrap cycles (95% CI 0.61, 0.75), demonstrating that the prediction results of the nomogram were consistent with the actual results. The 1-year, 3-year, and 5-year calibration curves in the calibration chart were found to be very close to the best predictive curve (45° line), indicating that the predictive performance of the nomogram on prognosis was good (**Figures 5C–E**).

**Figure 6:**
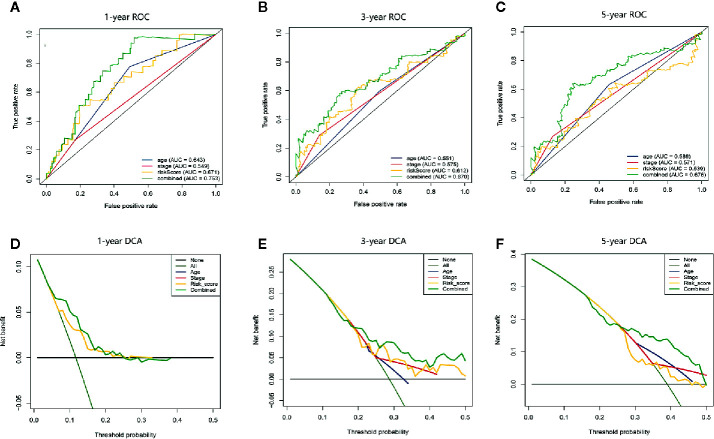
The time-dependent ROC and DCA curves were drawn to compare the predictive accuracy for prognosis between different nomograms. **(A–C)** Time-dependent ROC curve analysis validated the predictive accuracy of the nomograms constructed from different independent prognostic factors at 1 year, 3 years, and 5 years. **(D–F)** DCA visually assesses the clinical benefits of nomograms and the range of applications in which the nomogram can yield clinical benefit at 1 year, 3 years, and 5 years.

In addition, we compared the predictive accuracy between the nomogram and a single independent prognostic factor (age, clinical stage, or the apoptosis-related prognostic signature). We found that the C index of the nomogram (0.68) was superior to the C index of age (0.57), clinical stage (0.54), or prognostic model (0.66), confirming that the nomogram had better predictive value than any single independent prognostic factor. The ROC curve showed that the AUCs of the nomogram at 1 year, 3 years, and 5 years were all the largest (**Figures 6A–C**), demonstrating that the nomogram had the best predictive performance. The results of DCA also revealed that the nomogram provided the best net benefit compared to any single independent prognostic factor (**Figures 6D–F**). These findings demonstrate that the nomogram was more accurate than any single independent prognostic factor in predicting OS in HCC patients.

### Construction and Validation of the Diagnostic Model Based on Two Apoptosis Genes

**Figure 7:**
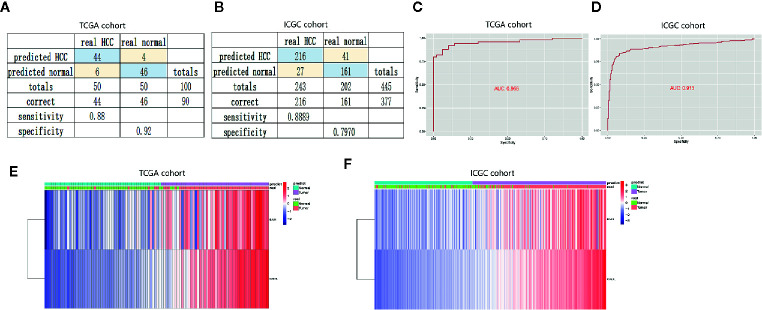
Construction and validation of diagnostic model to distinguish HCC from normal samples. **(A, B)** Confusion matrices of binary data showed high specificity and sensitivity of diagnostic model in the training cohort (TCGA cohort) and validation cohort (ICGC cohort). **(C, D)** Cox analysis and ROC curve predicted the good efficiency of diagnostic model both in TCGA cohort and ICGC cohort. **(E, F)** Unsupervised hierarchical clustering showed the expression of BAK1 and CSE1L in HCC and normal samples.

To confirm the diagnostic value of the two apoptosis-related genes, we established a diagnostic model to distinguish HCC and normal samples. The diagnostic score = −8.525 + (0.317* the expression level of BAK1) + (0.923* the expression level of CSE1L). According to the diagnostic model, the predicted specificity and sensitivity was 0.92 and 0.88 in the TCGA cohort (including 50 paired HCC and normal samples) (**Figure 7A**), and the specificity and sensitivity in the ICGC cohort (containing 243 HCC and 202 normal samples) was 0.80 and 0.89 (**Figure 7B**). ROC curve analysis showed the AUC was 0.956 in the TCGA cohort (**Figure 7C**) and 0.913 in the ICGC cohort (**Figure 7D**), respectively. Unsupervised hierarchical clustering indicated the two gene model separated HCC from normal samples with higher specificity and sensitivity (**Figures 7E, F**).

**Figure 8:**
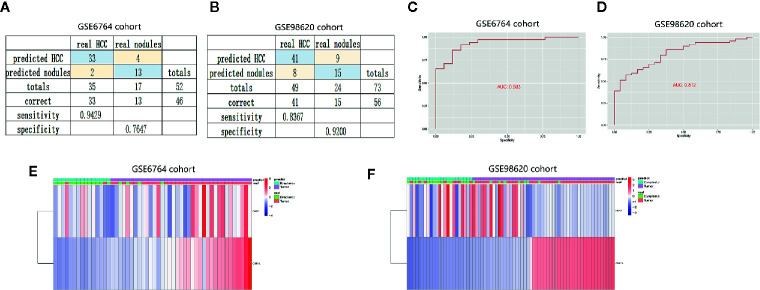
Establishment and validation of diagnostic model to distinguish HCC from dysplastic nodules. **(A, B)** Confusion matrices of binary data indicated high specificity and sensitivity of model to identify HCC and dysplastic nodules in training cohort (GSE6764 cohort) and validation cohort (GSE98620 cohort). **(C, D)** Cox analysis and ROC curve predicted the good efficiency of diagnostic model both in GSE98620 cohort and GSE6764 cohort. **(E, F)** Unsupervised hierarchical clustering indicated the level of BAK1 and CSE1L in HCC and dysplastic nodules.

Dysplastic nodules less than 2 cm in diameter were not well detected by CT or MRI. To further evaluate the diagnostic value of the two genes, we constructed diagnostic model to distinguish HCC from dysplastic nodules. The diagnostic score = −63.824 + (2.03* the expression level of BAK1) + (9.21* the expression level of CSE1L). The diagnostic model showed specificity at 0.76 and sensitivity at 0.94 in training cohort (GSE 6764) (**Figure 8A**), and specificity at 0.92 and sensitivity at 0.84 in validation (GSE 98620) (**Figure 8B**). The AUC of the diagnostic models were 0.933 and 0.812 in the two cohort (**Figures 8C, D**). Unsupervised hierarchical clustering showed the two gene models had good diagnostic value of distinguishing HCC from dysplastic nodules (**Figures 8E, F**). These data suggested two apoptotic genes were potential diagnostic markers for HCC.

### Further Validation of the Expression of Two Apoptosis Genes and Their Predictive Power for OS and RFS

**Figure 9:**
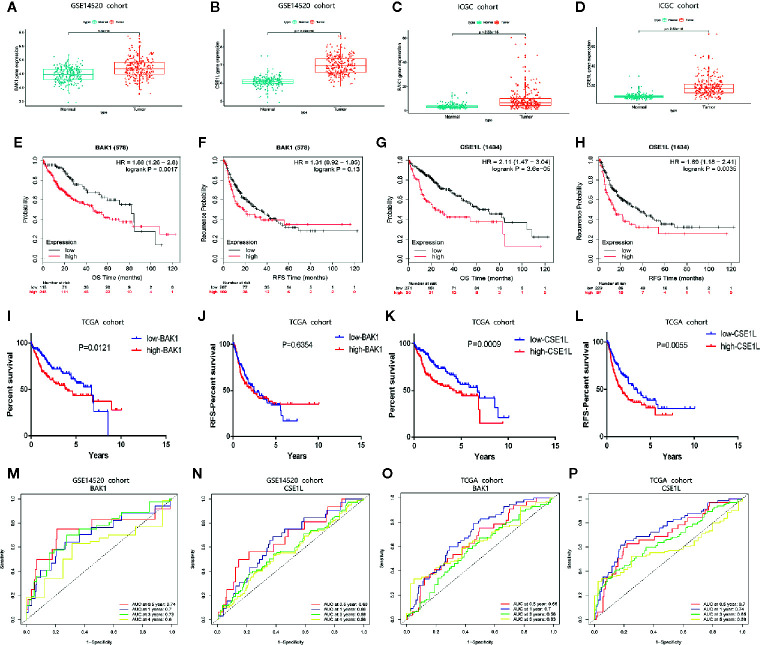
The expression patterns of BAK1 and CSE1L and their predictive power for OS and RFS. **(A**, **B)** Box plot indicating higher expression of BAK1 and CSE1L in HCC tissues (n = 243) than those in normal liver tissues (n = 241) in GSE14520. **(C**, **D)** Box plot indicating that expression of BAK1 and CSE1L in HCC tissues (n = 243) were significantly higher than those in normal liver tissues (n = 202) in ICGC. **(E–H)** Survival analysis of BAK1 and CSE1L in Kaplan-Meier Plotte database. **(I**, **J)** K-M curves indicating the predictive power of different expression of BAK1 for OS and RFS. **(K**, **L)** K-M curves indicating the predictive power of different expression of CSE1L for OS and RFS. **(M**, **N)** Time-dependent ROC curve analysis of the two apoptosis genes in the GSE14520 cohort. **(O**, **P)** Time-dependent ROC curve analysis of the two apoptosis genes in the TCGA cohort.

To further validate the value of the two apoptosis-related genes (BAK1 and CSE1L) for constructing the prognostic and diagnostic signatures, we adopted HCC cohorts from ICGC database and the GEO database (GSE14520) in to analyze the expression levels of these two apoptotic genes. The results of validation analysis in GSE14520 (**Figures 9A, B**) and ICGC (**Figures 9C, D**) are consistent with the finding in UALCAN database. Survival analysis in Kaplan-Meier Plotte (16) revealed that the OS of HCC patients with high expression of BAK1 was significantly lower than that of patients with low expression of BAK1 (P = 0.001) in TCGA cohort (**Figure 9E**) while there was no significant difference in RFS (P = 0.13) (**Figure 9F**). HCC patients with high CSE1L expression had significantly lower OS and RFS than patients with low expression of CSE1L (P < 0.05) (**Figures 9G, H**). K-M curves we performed in HCC cohort from TCGA is in accordance with the analysis result in Kaplan-Meier Plotte (**Figures 9I–L**). In the HCC cohort from GSE14520 and TCGA, The AUCs in time-dependent ROC curve analysis confirmed that BAK1 and CSE1L have great predictive value of prognosis (**Figures 9M–P**).

### Exploration of the Potential Mechanism of BAK1 and CSE1L in the HCC

**Figure 10:**
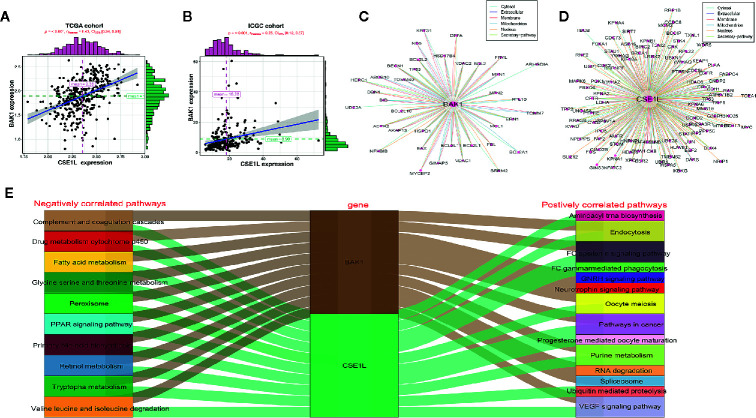
The potential regulatory mechanism of BAK1 and CES1L in the HCC. **(A, B)** Dependence Analysis showed BAK1 expression had positive correlation with CSE1L in the HCC in TCGA cohort and ICGC cohort. **(C, D)** PPI network showed the proteins interacted with BAK1 and CSE1L. **(E)** GSEA analysis indicated BAK1 and CSE1L regulated the underlying signaling pathways in the HCC and the top 10 signaling pathways were showed by Sankey diagram.

For exploring underlying mechanism of two apoptosis genes in HCC, correlation analysis was performed and indicated BAK1 expression had positive correlation with CSE1L in HCC in TCGA cohort and ICGC cohort (**Figures 10A, B**). PPI network showed the potential regulator which interacted with BAK1 and CSE1L (**Figures 10C, D**). In addition, GSEA analysis showed BAK1 and CSE1L regulated underlying signal pathway in HCC (**Tables S3**, **S4**). According to the top 10 signal pathway based on GSEA analysis, we found BAK1 and CSE1L also played positively role in signaling pathway which is relatively correlation with HCC including pathway in cancer. Moreover, BAK1 and CSE1L played a common role in all the top 10 negatively correlated pathways (**Figure 10E**). The results suggested BAK1 and CSE1L may played a synergistic role in the progression and development of hepatocellular carcinoma.

## Discussion

Due to the complex molecular pathogenesis, HCC is one of the most deadly malignant tumors in the world, with high mortality and recurrence rates and poor disease outcomes (1, 17). The recurrence of HCC is closely related to the early diagnosis and prognosis, but there is no consistent early diagnosis and prognostic examination standard so far. The diagnosis of HCC is rely on pathological (18) or radiological evaluation (19, 20), and the prognosis of HCC is mainly evaluated by AJCC TNM classification and BCLC staging currently (21). However, these methods of assessing the diagnosis and prognosis of HCC patients are far from satisfactory. Some studies have shown that a combination of molecular markers may have good value in evaluating the diagnosis and prognosis of cancer patients, which may be more helpful in predicting clinical outcomes (22–24). Realizing that biomolecular markers may provide a more effective predictive tool for tumor diagnosis and prognosis in clinical practice, comprehensive genomic research at the RNA level has received extensive attention. However, the number of molecular markers that have been identified as diagnosis and prognostic factors are still very small. In order to reduce the mortality and recurrence rates, improve clinical outcomes and survival, and help clinicians more effectively specify individualized treatment strategies, we urgently need to screen out new molecular markers that can predict diagnosis and prognosis more accurately.

Apoptosis, also identified as programmed cell death, is an important component of the physiological process to regulate the renewal or death of normal cells (9). However, dysregulation of apoptosis plays a critical role in the occurrence of many diseases and even multiple cancers. During the development and progression of cancer, alterations in the intracellular apoptotic pathway induce a reduction in apoptosis, and decreased apoptosis prevents malignant cells from dying, which promotes the development of cancer and is associated with resistance to treatment (25, 26). Correspondingly, apoptosis can also play an important role in cancer treatment as a therapeutic target, affecting the prognosis of tumors. Most of the current research has focused on the role of single apoptosis-related genes in the diagnosis and prognosis of HCC. Few studies have explored the association between apoptotic genes and the diagnosis and prognosis of HCC through high‐throughput sequencing of biomarkers.

In this study, we used high-throughput expression profiling of apoptotic genes to analyze the ability of apoptotic genes to predict prognosis in HCC patients. By comparing the differences in gene expression between HCC and adjacent non-tumor liver tissues in the TCGA database, we screened for differentially expressed apoptotic genes. Subsequently, univariate and multivariate Cox regression analyses were used to identify two apoptotic genes that were significantly associated with HCC prognosis: BAK1 and CSE1L. We thus constructed an apoptosis-related prognostic signature to predict HCC prognosis and survival. The K-M curve and ROC curve indicated that the two apoptotic genes had good prognostic performance. Further validation of the predictive accuracy of the prognostic signature was performed through GEO, and the expression levels of those two apoptotic genes were analyzed in GSE14520 cohort and ICGC cohort. We also demonstrated that the predictive prognostic ability of this apoptotic-related prognostic signature was independent of other clinical features of HCC by univariate and multivariate Cox regression analyses (P < 0.05). A nomogram can predict the probability of a clinical outcome (tumor recurrence or survival) for a given individual to help the clinician make clinical decisions and develop an individualized treatment plan for the patient (27). In this study, we integrated three independent prognostic factors of HCC identified by multivariate Cox regression analysis to construct a nomogram to accurately predict the prognosis and OS of HCC patients. The calibration curve indicated that the actual OS was closely related to the predicted survival rate, suggesting that the nomogram had good prognostic value.

Dysplastic nodules are premalignant neoplastic nodules usually occurred in cirrhotic livers, which divided into nodules (RNs), low grade dysplastic nodules (LGDN), high grade dysplastic nodules (HGDN), and finally small HCC (less than 2 cm). Identification of HCC during early stage is more likely to obtain longer survival time. Currently, the combination of radiological examination and pathological diagnosis is mainly used for early diagnosis of HCC, however, small nodules smaller than 2 cm are sometimes difficult to be characterized by pathology or radiology and are likely to be missed (28). The discrimination of dysplastic nodules is not easy based on uncertain clinical feature (29–31), since there are also morphological criteria used to define early HCC in well-differentiated dysplastic nodules (32). Sometimes it is difficult to distinguish the morphological criteria between dysplastic nodules and well-differentiated HCC. Base on the two apoptotic genes, we also constructed two diagnostic models to distinguish HCC from normal samples and dysplastic nodules. In the study, Cox analysis and ROC curve indicated the diagnostic model have good efficiency with high specificity and sensitivity. The great diagnostic performance of the diagnostic model may help detect early small HCC missed by imaging and pathological diagnosis.

CSE1L (chromosome-isolated protein), also known as CAS (apoptosis-susceptible protein), is a multifunctional protein involved in multiple physiological and pathological processes, such as cell survival, apoptosis, nuclear cytoplasmic migration, microencapsulation, and hyperplasia and migration of cancer cells (33). CSE1L/CAS is overexpressed in multiple types of cancers, and many pathological reports have revealed that the expression level of CSE1L is closely associated with cancer proliferation and metastasis (34, 35). BAK1 (BCL2 antagonist/killer 1) is a key regulator of mitochondria-mediated apoptosis (36). The protein encoded by BAK1 belongs to the BCL2 protein family. There has been no research on the association between BAK1 and CSE1L in the diagnosis and prognosis of HCC. BAK1 and CSE1L inhibition suppressed HCC cells proliferation and promoted HCC cells apoptosis. BAK1 and CSE1L may be regard as oncogenic regulators of HCC. Correlation analysis indicated the two genes had positive correlation in HCC. BAK1 and CSE1L also interacted with the same genes including P53 and they regulated same signaling pathway including pathway in cancer, PPAR signaling pathways, etc. That means BAK1 and CSE1L may played synergistic role in the progression and development in the HCC.

Inevitably, our current research has some limitations. First, our study is based on RNA-sequencing data from the public dataset TCGA, and without prospective testing in clinical trials, the predictive performance of the apoptosis-related prognostic signature and nomogram requires further validation in multicenter clinical trials and prospective studies. In addition, the oncogenic effect of BAK1 and CSE1L of HCC need to be elucidated and the underlying mechanism remain to be further explored.

## Conclusion

In conclusion, we identified the prognostic signature of two apoptosis-related genes as a reliable tool for more accurate prediction of diagnosis and prognosis in HCC patients, and the diagnostic models to distinguish HCC from normal samples/proliferative nodules (≦2cm) with high specificity and sensitivity, which may help doctors to develop more effective early diagnostic and individualized treatment strategies.

## Data Availability Statement

Publicly available datasets were analyzed in this study; these can be found in The Cancer Genome Atlas (https://www.tcga.org/) and Deathbase (http://www.deathbase.org/).

## Author Contributions

JJ and MX conceived and designed the experiments. BT and JZ performed the experiments. XL, MM, QW, and NZ analyzed the data. JL, KF, LZ, and SF contributed analysis tools. BT and JZ wrote the paper. JJ and MX edited the paper. All authors contributed to the article and approved the submitted version.

## Funding

This study was supported by National Natural Science Foundation of China (Nos. 81803778), and The Key Research and development Project of Zhejiang Province (No. 2018C0302), and Public Welfare Project of Zhejiang Province (Nos. LGF19H180010, LGD19H160002, and LGF19H180009), and Medical and Health Care Key Project of Zhejiang Province (Nos. 2016146810, 2018KY197, and 2018KY932).

## Conflict of Interest

The authors declare that the research was conducted in the absence of any commercial or financial relationships that could be construed as a potential conflict of interest.

## Supplementary Material

Supplementary Figure 1 Functional enrichment analysis of 14 differentially expressed apoptotic genes between HCC tissues and non-cancerous tissues. **(A)** The GO function enrichment analysis of the 14 apoptosis genes. **(B)** The KEGG pathway concentrating on the 14 apoptosis genes.

Supplementary Figure 2 Distribution of risk score in HCC patients and the survival status of the patients. **(A, C, E)** HCC patients were divided into high-risk group and low-risk group according to the level of risk index in the TCGA cohort **(A)**, GSE14520 cohort **(C)**, and ICGC cohort. **(B, D, F)** The heat map shows the relationship between different risk scores and the expression levels of BAK1 and CSE1L in the TCGA cohort **(B)**, GSE14520 cohort **(D)**, and ICGC cohort **(F)**.

Supplementary Table 1 Apoptosis genes extracted from the Deathbase.

Supplementary Table 2 Univariate and multivariate Cox regression analyses of the prognostic signature and clinical features related to OS in HCC patients.

Supplementary Table 3 Underlying signal pathway in HCC regulated by BAK1 determined by GSEA analysis

Supplementary Table 4 Underlying signal pathway in HCC regulated by CSE1L determined by GSEA analysis
