# Supplementary figures and images for: Identifying Apoptosis-Related Transcriptomic Aberrations and Revealing Clinical Relevance as Diagnostic and Prognostic Biomarker in Hepatocellular Carcinoma
Source: Front Oncol. 2021 Feb 18;10:519180. doi: 10.3389/fonc.2020.519180 (PMC7931692; doi:10.3389/fonc.2020.519180)

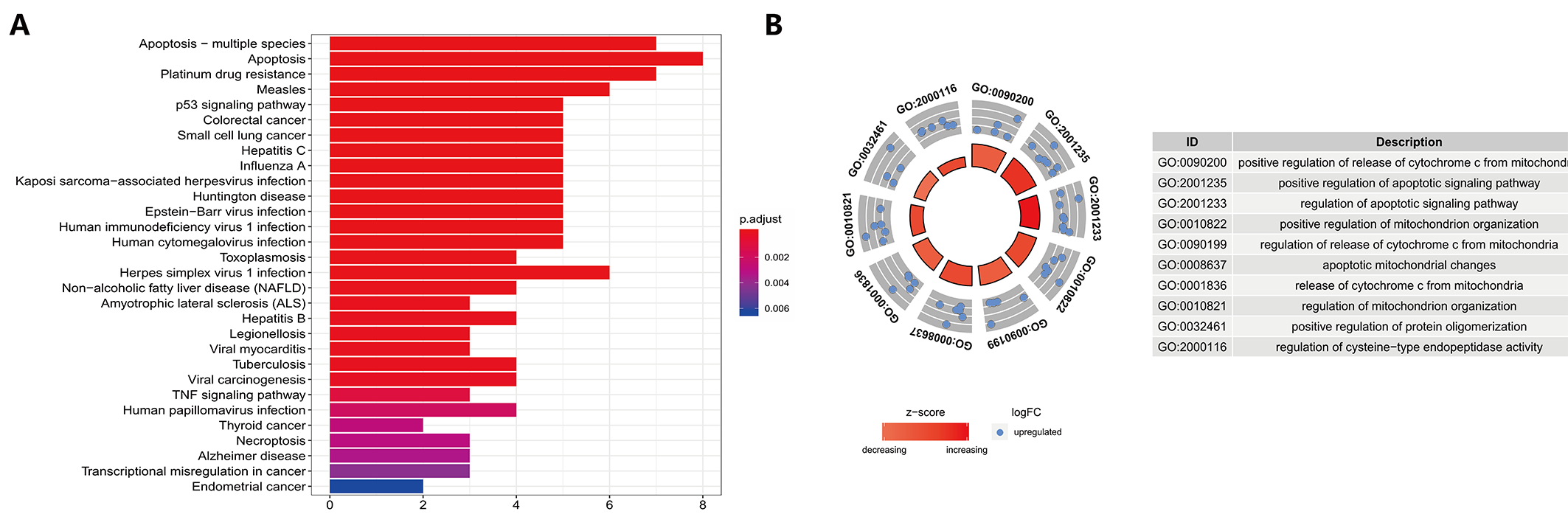

Supplement: Supplementary Figure 1 — Functional enrichment analysis of 14 differentially expressed apoptotic genes between HCC tissues and non-cancerous tissues. (A) The GO function enrichment analysis of the 14 apoptosis genes. (B) The KEGG pathway concentrating on the 14 apoptosis genes. [file Image_1.tif]

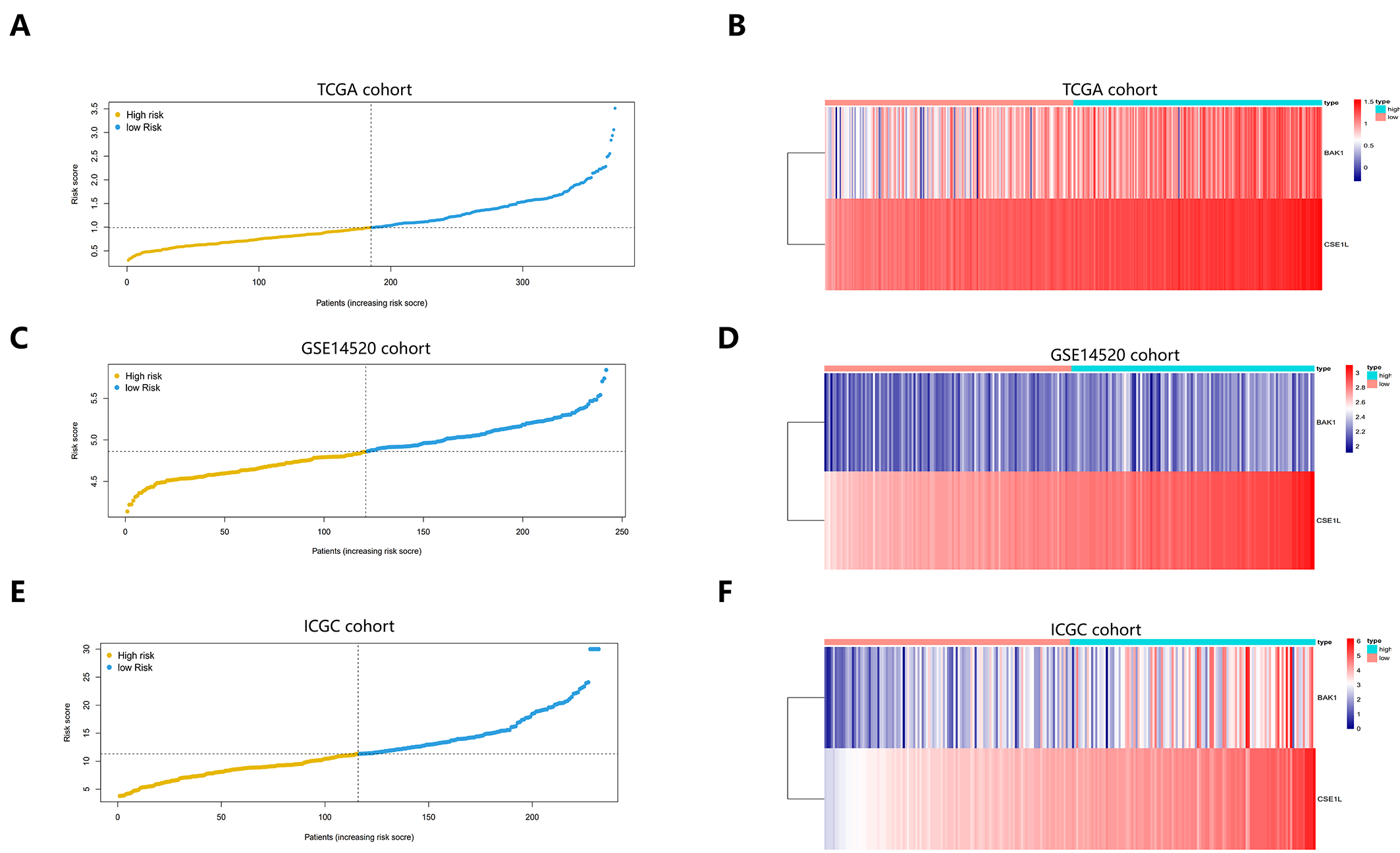

Supplement: Supplementary Figure 2 — Distribution of risk score in HCC patients and the survival status of the patients. (A, C, E) HCC patients were divided into high-risk group and low-risk group according to the level of risk index in the TCGA cohort (A), GSE14520 cohort (C), and ICGC cohort. (B, D, F) The heat map shows the relationship between different risk scores and the expression levels of BAK1 and CSE1L in the TCGA cohort (B), GSE14520 cohort (D), and ICGC cohort (F). [file Image_2.tif]
