# Supplementary material for: Identifying Apoptosis-Related Transcriptomic Aberrations and Revealing Clinical Relevance as Diagnostic and Prognostic Biomarker in Hepatocellular Carcinoma
Source: Front Oncol. 2021 Feb 18;10:519180. doi: 10.3389/fonc.2020.519180 (PMC7931692; doi:10.3389/fonc.2020.519180)
Supplement: Supplementary Table 1 — Apoptosis genes extracted from the Deathbase. [file Table_1.docx]

| **ensembl_gene_id** | **external_id** | **synonyms** | **description** |
| --- | --- | --- | --- |
| ENSG00000140350 | ANP32A | LANP, PP32, I1PP2A, PHAPI, MAPM, mapmodulin | Acidic leucine-rich nuclear phosphoprotein 32 family member A (Potent heat-stable protein phosphatase 2A inhibitor I1PP2A)(Acidic nuclear phosphoprotein pp32)(Leucine-rich acidic nuclear protein)(Lanp)(Putative HLA-DR-associated protein I)(PHAPI)(Mapmodulin) [Source:UniProtKB/Swiss-Prot;Acc:P39687] |
| ENSG00000120868 | APAF1 | CED4 | Apoptotic protease-activating factor 1 (Apaf-1) [Source:UniProtKB/Swiss-Prot;Acc:O14727] |
| ENSG00000169857 | AVEN | PDCD12 | Cell death regulator Aven [Source:UniProtKB/Swiss-Prot;Acc:Q9NQS1] |
| ENSG00000002330 | BAD | BCL2L8, BBC2 | Bcl2 antagonist of cell death (BAD)(Bcl-2-binding component 6)(Bcl-XL/Bcl-2-associated death promoter)(Bcl-2-like 8 protein) [Source:UniProtKB/Swiss-Prot;Acc:Q92934] |
| ENSG00000030110 | BAK2 | BAK2 | Bcl-2 homologous antagonist/killer (Apoptosis regulator BAK)(Bcl-2-like 7 protein) [Source:UniProtKB/Swiss-Prot;Acc:Q16611] |
| ENSG00000030110 | BAK1 | BCL2L7, BAK | Bcl-2 homologous antagonist/killer (Apoptosis regulator BAK)(Bcl-2-like 7 protein) [Source:UniProtKB/Swiss-Prot;Acc:Q16611] |
| ENSG00000087088 | BAX | BCL2L4 | Apoptosis regulator BAX [Source:UniProtKB/Swiss-Prot;Acc:Q07812] |
| ENSG00000105327 | BBC3 | JFY1, PUMA | Bcl-2-binding component 3 (p53 up-regulated modulator of apoptosis)(JFY-1) [Source:UniProtKB/Swiss-Prot;Acc:Q9BXH1] |
| ENSG00000171791 | BCL2 | Bcl-2 | Apoptosis regulator Bcl-2 [Source:UniProtKB/Swiss-Prot;Acc:P10415] |
| ENSG00000140379 | BCL2A1 | GRS, BFL1, BCL2L5, ACC-1, ACC-2 | Bcl-2-related protein A1 (Protein BFL-1)(Hemopoietic-specific early response protein)(Protein GRS) [Source:UniProtKB/Swiss-Prot;Acc:Q16548] |
| ENSG00000171552 | BCL2L1 | BCLX, BCL2L, Bcl-X, bcl-xL, bcl-xS | Apoptosis regulator Bcl-X (Bcl-2-like 1 protein) [Source:UniProtKB/Swiss-Prot;Acc:Q07817] |
| ENSG00000137875 | BCL2L10 | Diva, Boo, BCL-B | Apoptosis regulator Bcl-B (Bcl-2-like 10 protein)(Bcl2-L-10)(Anti-apoptotic protein NrH) [Source:UniProtKB/Swiss-Prot;Acc:Q9HD36] |
| ENSG00000153094 | BCL2L11 | BOD, BimL, BimEL, BIM | Bcl-2-like protein 11 (Bcl2-interacting mediator of cell death) [Source:UniProtKB/Swiss-Prot;Acc:O43521] |
| ENSG00000126453 | BCL2L12 |  | Bcl-2-related proline-rich protein (Bcl-2-like 12 protein) [Source:UniProtKB/Swiss-Prot;Acc:Q9HB09] |
| ENSG00000121380 | BCL2L14 | BCLG, BCL-G | Apoptosis facilitator Bcl-2-like 14 protein (Apoptosis regulator Bcl-G) [Source:UniProtKB/Swiss-Prot;Acc:Q9BZR8] |
| ENSG00000129473 | BCL2L2 | KIAA0271, BCL-W | Apoptosis regulator Bcl-W (Bcl-2-like 2 protein) [Source:UniProtKB/Swiss-Prot;Acc:Q92843] |
| ENSG00000015475 | BID |  | BH3-interacting domain death agonist (p22 BID)(BID) [Contains BH3-interacting domain death agonist p15(p15 BID);BH3-interacting domain death agonist p13(p13 BID);BH3-interacting domain death agonist p11(p11 BID)] [Source:UniProtKB/Swiss-Prot;Acc:P55957] |
| ENSG00000100290 | BIK | NBK | Bcl-2-interacting killer (Apoptosis inducer NBK)(BP4)(BIP1) [Source:UniProtKB/Swiss-Prot;Acc:Q13323] |
| ENSG00000110330 | BIRC2 | cIAP1, hiap-2, MIHB, RNF48 | Baculoviral IAP repeat-containing protein 2 (Inhibitor of apoptosis protein 2)(HIAP-2)(HIAP2)(C-IAP1)(TNFR2-TRAF-signaling complex protein 2)(IAP homolog B)(RING finger protein 48) [Source:UniProtKB/Swiss-Prot;Acc:Q13490] |
| ENSG00000023445 | BIRC3 | cIAP2, hiap-1, MIHC, RNF49, MALT2 | Baculoviral IAP repeat-containing protein 3 (Inhibitor of apoptosis protein 1)(HIAP-1)(HIAP1)(C-IAP2)(TNFR2-TRAF-signaling complex protein 1)(IAP homolog C)(Apoptosis inhibitor 2)(API2)(RING finger protein 49) [Source:UniProtKB/Swiss-Prot;Acc:Q13489] |
| ENSG00000104081 | BMF | FLJ00065 | Bcl-2-modifying factor [Source:UniProtKB/Swiss-Prot;Acc:Q96LC9] |
| ENSG00000176171 | BNIP3 | Nip3 | BCL2/adenovirus E1B 19 kDa protein-interacting protein 3 [Source:UniProtKB/Swiss-Prot;Acc:Q12983] |
| ENSG00000104765 | BNIP3L | Nix, BNIP3a | BCL2/adenovirus E1B 19 kDa protein-interacting protein 3-like (NIP3-like protein X)(NIP3L)(BCL2/adenovirus E1B 19 kDa protein-interacting protein 3A)(Adenovirus E1B19K-binding protein B5) [Source:UniProtKB/Swiss-Prot;Acc:O60238] |
| ENSG00000003400 | CASP10 | MCH4 | Caspase-10 Precursor (CASP-10)(EC 3.4.22.63)(ICE-like apoptotic protease 4)(Apoptotic protease Mch-4)(FAS-associated death domain protein interleukin-1B-converting enzyme 2)(FLICE2) [Contains Caspase-10 subunit p23/17;Caspase-10 subunit p12] [Source:UniProtKB/Swiss-Prot;Acc:Q92851] |
| ENSG00000164305 | CASP3 | CPP32, CPP32B, Yama, apopain | Caspase-3 Precursor (CASP-3)(EC 3.4.22.56)(Apopain)(Cysteine protease CPP32)(CPP-32)(Yama protein)(SREBP cleavage activity 1)(SCA-1) [Contains Caspase-3 subunit p17;Caspase-3 subunit p12] [Source:UniProtKB/Swiss-Prot;Acc:P42574] |
| ENSG00000138794 | CASP6 | MCH2 | Caspase-6 Precursor (CASP-6)(EC 3.4.22.59)(Apoptotic protease Mch-2) [Contains Caspase-6 subunit p18;Caspase-6 subunit p11] [Source:UniProtKB/Swiss-Prot;Acc:P55212] |
| ENSG00000165806 | CASP7 | MCH3, CMH-1, ICE-LAP3 | Caspase-7 Precursor (CASP-7)(EC 3.4.22.60)(ICE-like apoptotic protease 3)(ICE-LAP3)(Apoptotic protease Mch-3)(CMH-1) [Contains Caspase-7 subunit p20;Caspase-7 subunit p11] [Source:UniProtKB/Swiss-Prot;Acc:P55210] |
| ENSG00000064012 | CASP8 | MCH5, MACH, FLICE | Caspase-8 Precursor (CASP-8)(EC 3.4.22.61)(ICE-like apoptotic protease 5)(MORT1-associated CED-3 homolog)(MACH)(FADD-homologous ICE/CED-3-like protease)(FADD-like ICE)(FLICE)(Apoptotic cysteine protease)(Apoptotic protease Mch-5)(CAP4) [Contains Caspase-8 subunit p18;Caspase-8 subunit p10] [Source:UniProtKB/Swiss-Prot;Acc:Q14790] |
| ENSG00000132906 | CASP9 | MCH6, ICE-LAP6, APAF-3 | Caspase-9 Precursor (CASP-9)(EC 3.4.22.62)(ICE-like apoptotic protease 6)(ICE-LAP6)(Apoptotic protease Mch-6)(Apoptotic protease-activating factor 3)(APAF-3) [Contains Caspase-9 subunit p35;Caspase-9 subunit p10] [Source:UniProtKB/Swiss-Prot;Acc:P55211] |
| ENSG00000003402 | CFLAR | CASH, Casper, CLARP, FLAME, FLIP, I-FLICE, MRIT | CASP8 and FADD-like apoptosis regulator Precursor (Cellular FLICE-like inhibitory protein)(c-FLIP)(Caspase-eight-related protein)(Casper)(Caspase-like apoptosis regulatory protein)(CLARP)(MACH-related inducer of toxicity)(MRIT)(Caspase homolog)(CASH)(Inhibitor of FLICE)(I-FLICE)(FADD-like antiapoptotic molecule 1)(FLAME-1)(Usurpin) [Contains CASP8 and FADD-like apoptosis regulator subunit p43;CASP8 and FADD-like apoptosis regulator subunit p12] [Source:UniProtKB/Swiss-Prot;Acc:O15519] |
| ENSG00000169372 | CRADD | RAIDD | Death domain-containing protein CRADD (Caspase and RIP adapter with death domain)(RIP-associated protein with a death domain) [Source:UniProtKB/Swiss-Prot;Acc:P78560] |
| ENSG00000124207 | CSE1L | CAS, XPO2, CSE1 | Exportin-2 (Exp2)(Importin-alpha re-exporter)(Chromosome segregation 1-like protein)(Cellular apoptosis susceptibility protein) [Source:UniProtKB/Swiss-Prot;Acc:P55060] |
| ENSG00000172115 | CYCS | HCS | Cytochrome c [Source:UniProtKB/Swiss-Prot;Acc:P99999] |
| ENSG00000184047 | DIABLO | SMAC, DIABLO-S, FLJ25049, FLJ10537 | Diablo homolog, mitochondrial Precursor (Second mitochondria-derived activator of caspase)(Smac protein)(Direct IAP-binding protein with low pI) [Source:UniProtKB/Swiss-Prot;Acc:Q9NR28] |
| ENSG00000168040 | FADD | MORT1, GIG3 | Protein FADD (FAS-associated death domain protein)(FAS-associating death domain-containing protein)(Mediator of receptor induced toxicity) [Source:UniProtKB/Swiss-Prot;Acc:Q13158] |
| ENSG00000026103 | FAS | CD95, APO-1 | Tumor necrosis factor receptor superfamily member 6 Precursor (FASLG receptor)(Apoptosis-mediating surface antigen FAS)(Apo-1 antigen)(CD95 antigen) [Source:UniProtKB/Swiss-Prot;Acc:P25445] |
| ENSG00000117560 | FASLG | FasL, CD178 | Tumor necrosis factor ligand superfamily member 6 (Fas antigen ligand)(Fas ligand)(CD95L protein)(Apoptosis antigen ligand)(APTL)(CD178 antigen) [Contains Tumor necrosis factor ligand superfamily member 6, membrane form;Tumor necrosis factor ligand superfamily member 6, soluble form] [Source:UniProtKB/Swiss-Prot;Acc:P48023] |
| ENSG00000135116 | HRK | DP5 | Activator of apoptosis harakiri (Neuronal death protein DP5)(BH3-interacting domain-containing protein 3) [Source:UniProtKB/Swiss-Prot;Acc:O00198] |
| ENSG00000143384 | MCL1 | BCL2L3 | Induced myeloid leukemia cell differentiation protein Mcl-1 (Bcl-2-related protein EAT/mcl1)(mcl1/EAT) [Source:UniProtKB/Swiss-Prot;Acc:Q07820] |
| ENSG00000165943 | MOAP1 | MAP-1, PNMA4 | Modulator of apoptosis 1 (MAP-1)(MAP1)(Paraneoplastic antigen Ma4) [Source:UniProtKB/Swiss-Prot;Acc:Q96BY2] |
| ENSG00000141682 | PMAIP1 | APR, NOXA | Phorbol-12-myristate-13-acetate-induced protein 1 (PMA-induced protein 1)(Immediate-early-response protein APR)(NOXA) [Source:UniProtKB/Swiss-Prot;Acc:Q13794] |
| ENSG00000137275 | RIPK1 | RIP | Receptor-interacting serine/threonine-protein kinase 1 (EC 2.7.11.1)(Serine/threonine-protein kinase RIP)(Cell death protein RIP)(Receptor-interacting protein) [Source:UniProtKB/Swiss-Prot;Acc:Q13546] |
| ENSG00000097033 | SH3GLB1 | CGI-61, KIAA0491, Bif-1 | Endophilin-B1 (SH3 domain-containing GRB2-like protein B1)(Bax-interacting factor 1)(Bif-1) [Source:UniProtKB/Swiss-Prot;Acc:Q9Y371] |
| ENSG00000204490 | TNF | TNFSF2, DIF, TNF-alpha | Tumor necrosis factor Precursor (TNF-alpha)(Tumor necrosis factor ligand superfamily member 2)(TNF-a)(Cachectin) [Contains Tumor necrosis factor, membrane form;Tumor necrosis factor, soluble form] [Source:UniProtKB/Swiss-Prot;Acc:P01375] |
| ENSG00000104689 | TNFRSF10A | DR4, Apo2, TRAILR-1, CD261 | Tumor necrosis factor receptor superfamily member 10A Precursor (Death receptor 4)(TNF-related apoptosis-inducing ligand receptor 1)(TRAIL receptor 1)(TRAIL-R1)(CD261 antigen) [Source:UniProtKB/Swiss-Prot;Acc:O00220] |
| ENSG00000120889 | TNFRSF10B | DR5, KILLER, TRICK2A, TRAIL-R2, TRICKB, CD262 | Tumor necrosis factor receptor superfamily member 10B Precursor (Death receptor 5)(TNF-related apoptosis-inducing ligand receptor 2)(TRAIL receptor 2)(TRAIL-R2)(CD262 antigen) [Source:UniProtKB/Swiss-Prot;Acc:O14763] |
| ENSG00000173535 | TNFRSF10C | DcR1, TRAILR3, LIT, TRID, CD263 | Tumor necrosis factor receptor superfamily member 10C Precursor (Decoy receptor 1)(DcR1)(Decoy TRAIL receptor without death domain)(TNF-related apoptosis-inducing ligand receptor 3)(TRAIL receptor 3)(TRAIL-R3)(Trail receptor without an intracellular domain)(Lymphocyte inhibitor of TRAIL)(Antagonist decoy receptor for TRAIL/Apo-2L)(CD263 antigen) [Source:UniProtKB/Swiss-Prot;Acc:O14798] |
| ENSG00000173530 | TNFRSF10D | DcR2, TRUNDD, TRAILR4, CD264 | Tumor necrosis factor receptor superfamily member 10D Precursor (Decoy receptor 2)(DcR2)(TNF-related apoptosis-inducing ligand receptor 4)(TRAIL receptor 4)(TRAIL-R4)(TRAIL receptor with a truncated death domain)(CD264 antigen) [Source:UniProtKB/Swiss-Prot;Acc:Q9UBN6] |
| ENSG00000067182 | TNFRSF1A | TNF-R, TNFAR, TNFR60, TNF-R-I, CD120a, TNF-R55 | Tumor necrosis factor receptor superfamily member 1A Precursor (p60)(TNF-R1)(TNF-RI)(TNFR-I)(p55)(CD120a antigen) [Contains Tumor necrosis factor receptor superfamily member 1A, membrane form;Tumor necrosis factor-binding protein 1(TBPI)] [Source:UniProtKB/Swiss-Prot;Acc:P19438] |
| ENSG00000028137 | TNFRSF1B | TNFBR, TNFR80, TNF-R75, TNF-R-II, p75, CD120b | Tumor necrosis factor receptor superfamily member 1B Precursor (Tumor necrosis factor receptor 2)(TNF-R2)(Tumor necrosis factor receptor type II)(p75)(p80 TNF-alpha receptor)(CD120b antigen)(Etanercept) [Contains Tumor necrosis factor receptor superfamily member 1b, membrane form;Tumor necrosis factor-binding protein 2(TBPII)(TBP-2)] [Source:UniProtKB/Swiss-Prot;Acc:P20333] |
| ENSG00000121858 | TNFSF10 | TRAIL, Apo-2L, TL2, CD253 | Tumor necrosis factor ligand superfamily member 10 (TNF-related apoptosis-inducing ligand)(Protein TRAIL)(Apo-2 ligand)(Apo-2L)(CD253 antigen) [Source:UniProtKB/Swiss-Prot;Acc:P50591] |
| ENSG00000141510 | TP53 | p53, LFS1 | Cellular tumor antigen p53 (Tumor suppressor p53)(Phosphoprotein p53)(Antigen NY-CO-13) [Source:UniProtKB/Swiss-Prot;Acc:P04637] |
| ENSG00000102871 | TRADD | Hs.89862 | Tumor necrosis factor receptor type 1-associated DEATH domain protein (TNFR1-associated DEATH domain protein)(TNFRSF1A-associated via death domain) [Source:UniProtKB/Swiss-Prot;Acc:Q15628] |
| ENSG00000101966 | XIAP | hILP | Baculoviral IAP repeat-containing protein 4 (EC 6.3.2.-)(E3 ubiquitin-protein ligase XIAP)(Inhibitor of apoptosis protein 3)(X-linked inhibitor of apoptosis protein)(X-linked IAP)(IAP-like protein)(HILP) [Source:UniProtKB/Swiss-Prot;Acc:P98170] |
