# Supplementary material for: Identifying Apoptosis-Related Transcriptomic Aberrations and Revealing Clinical Relevance as Diagnostic and Prognostic Biomarker in Hepatocellular Carcinoma
Source: Front Oncol. 2021 Feb 18;10:519180. doi: 10.3389/fonc.2020.519180 (PMC7931692; doi:10.3389/fonc.2020.519180)
Supplement: Supplementary Table 2 — Univariate and multivariate Cox regression analyses of the prognostic signature and clinical features related to OS in HCC patients. [file Table_2.docx]

Table S2: Univariate and multivariate Cox regression analyses of the prognostic signature and clinical features related to OS in HCC patients.

| Characteristics | Univariate analysis | | Multivariate analysis | |
| --- | --- | --- | --- | --- |
|  | HR (95%CI) | P-value | HR (95%CI) | P-value |
| age（≥60/<60） | 1.686576 | 0.048041 | 1.907523 | 0.021804 |
| gender(Male/Female) | 0.649394 | 0.096388 |  |  |
| weight(64-189) | 0.988892 | 0.466736 |  |  |
| vascular tumor cell(yes/no) | 1.567373 | 0.093812 |  |  |
| AFP(≥300/<300) | 1.076633 | 0.797748 |  |  |
| Histologic grade(G3+G4/G1+G2) | 1.539132 | 0.091867 |  |  |
| Pathologic stage(III+IV/I+II) | 1.946615 | 0.014638 | 1.977811 | 0.019362 |
| riskScore(high risk group/low risk group) | 2.091724 | 0.001551 | 2.050495 | 0.006671 |
